# Multiple checkpoints ensure ribosomes have the correct end

**DOI:** 10.1371/journal.pbio.3002603

**Published:** 2024-04-30

**Authors:** Jacob Gordon, Robin E. Stanley

**Affiliations:** 1 Signal Transduction Laboratory, National Institute of Environmental Health Sciences, National Institutes of Health, Department of Health and Human Services, Research Triangle Park, Durham, North Carolina, United States of America; 2 Cambridge Institute for Medical Research, Cambridge, United Kingdom; 3 Department of Haematology, University of Cambridge, Cambridge, United Kingdom; 4 Wellcome Trust-Medical Research Council Stem Cell Institute, University of Cambridge, Cambridge, United Kingdom

## Abstract

The 3’ end of 18S ribosomal RNA is formed by the endoribonuclease Nob1, but how cells ensure the accuracy of the 3’ end has been a mystery. A new study in PLOS Biology has revealed multiple checkpoints that ensure only ribosomes containing the correct 3’ end participate in translation.

## Teaser

The 3′ end of the 18S ribosomal RNA is formed by the endoribonuclease Nob1. How cells ensure the accuracy of the 3′ end has remained a mystery. A new study in *PLOS Biology* revealed that there are multiple checkpoints to ensure that only ribosomes containing the correct 3′ end participate in translation.

Ribosome assembly is a complex and highly regulated process that relies on hundreds of assembly factors, including many ribonucleases which process the pre-ribosomal RNA (rRNA) [1]. The final pre-rRNA processing step of the 40S small subunit (SSU) is the cleavage of the 3′ end of the 20S pre-rRNA by the endoribonuclease Nob1. Cleavage by Nob1 removes the last fragment of the Internal Transcribed Spacer 1 (ITS1) and forms the mature 18S rRNA. Nob1 and its binding partner Pno1 (also known as Dim2) are recruited to nascent 40S subunits and shield these pre-40S particles from premature binding of mRNA and the ribosomal protein Rps26. Cryo-EM structures of pre-40S particles established that Pno1 also masks the ITS1 cleavage site from Nob1 and that conformational rearrangements are required for Nob1 cleavage [2–4]. Prior work established that Nob1 can miscleave rRNA in vitro [5]. While proper formation of the 3′ end is critically important, it has remained unclear how cells ensure the fidelity of this processing step.

In this issue of *PLOS Biology*, Parker and colleagues set out to answer this critical question through a series of experiments in *Saccharomyces cerevisiae* [6]. 3′ end sequencing revealed that Nob1 can miscleave its substrate in vivo. However, 98% of the 18S rRNAs have the correct end. To determine the consequence of improper 18S formation, an RNA Pol II-driven plasmid system was used to generate miscleaved 18S rRNA with truncated ends. Truncation of 2 to 4 nucleotides from the 3′ end impairs cells growth and slows down translation. Interestingly, the observation was made that these miscleaved ribosomes lead to ribosome collisions during translation. Ribosome collisions can form in vivo for a variety of reasons such as mRNA stall sequences, mRNA roadblocks, rare codons, or tRNA availability, and there are multiple quality control pathways that have been discovered that can detect these collided ribosomes [7]. Additional experiments confirmed that miscleaved 18S ribosomes rely on several factors important for ribosome-associated quality control (RQC) to promote their decay. These findings reveal that ribosome collisions provide a post-assembly checkpoint to remove ribosomes containing miscleaved 18S ends from the translation pool.

Parker and colleagues hypothesized that there must be a quality control checkpoint surveying the 18S end during assembly given that the occurrence of miscleaved 18S 3′ ends is rare in vivo, but not in vitro [6]. The ATPase Rio1 was a likely candidate for this checkpoint role because it is important for late stage 40S maturation [8]. Rio1 associates with rRNA helix 44 (h44) of the 18S rRNA located near the 3′ end and through conformational rearrangements, it triggers the release of Pno1 and Nob1 from pre-40S particles [2–4]. To determine if Rio1 functions as a quality control factor, Parker and colleagues carried out in vitro RNA-binding assays which revealed that Rio1 binds to a mimic of h44 with the correct end stronger than to h44 mimics with miscleaved ends. Finally, Parker and colleagues demonstrated that both bypassing Rio1 and overexpression of Rio1 disrupts late-stage assembly leading to the accumulation of miscleaved ribosomes [6]. Together, this work establishes that Rio1 plays a critical role in ensuring the accuracy of the 18S end during ribosome assembly.

**Fig 1:**
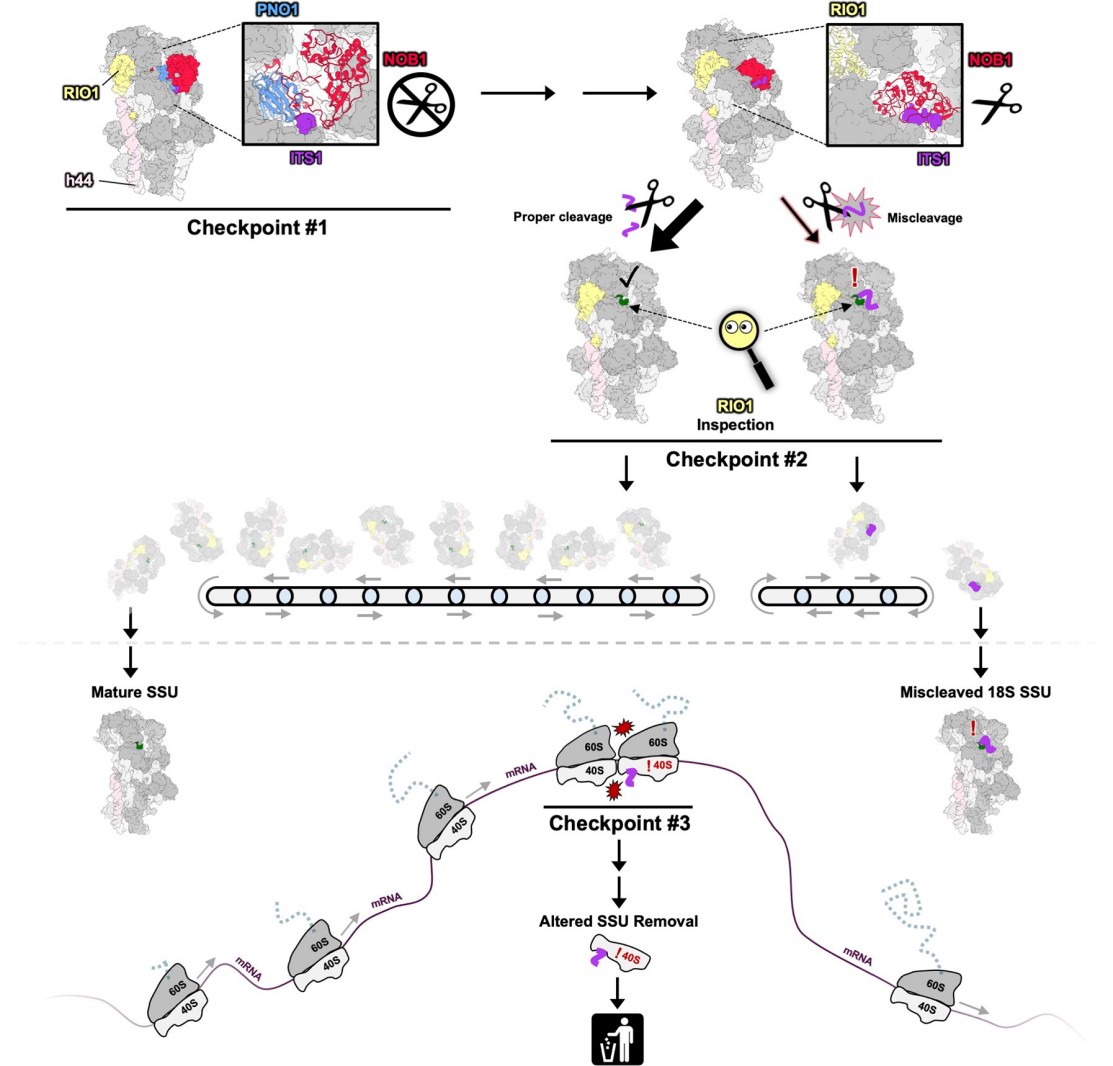
Multiple checkpoints ensure the fidelity of the 3′ end of the 18S rRNA. The nuclease Nob1 (red) and its binding partner Pno1 (blue) are recruited to pre-40S particles and bind near h44 (pink). Pno1 provides the first checkpoint for 3′ end formation by preventing premature cleavage by Nob1 through its shielding of the ITS1 cleavage site (purple). The ATPase Rio1 (yellow) triggers conformational changes that activate Nob1 cleavage and trigger the dissociation of Nob1 and Pno1 from the pre-40S. Rio1 promotes a second checkpoint by inspecting the 3′ end of the 18S rRNA (green = correct end, purple = miscleaved end) to prevent miscleaved ribosomes from entering the translating pool. A small percentage of miscleaved ribosomes escape the Rio1 checkpoint and enter the translating pool. These miscleaved ribosomes lead to ribosome collision-mediated decay, providing a third checkpoint that removes miscleaved ribosomes from the cell. The following coordinates were used to create the figure: pre-40S (PDBID: 6ZXE, 6ZXF, 6ZXG) [4] and mature 40S (PDBID: 7R4X) [10].

This work adds to the growing list of quality control check mechanisms that have been discovered which help ensure that ribosomes are made correctly to prevent downstream errors in translation [8]. Proper 18S 3′ end formation is safeguarded from potential Nob1 errors by at least 3 different quality control checkpoints (Fig 1). First, the assembly factor Pno1 acts as steric block to prevent premature cleavage of the ITS1 by Nob1. Second, Rio1 inspects the 3′ end to prevent the release of miscleaved ribosomes into the translating pool. Third, in the rare instance in which these miscleaved ribosomes enter the translating pool, their altered rates of translation promote ribosome-collision mediated decay.

In addition to Nob1, many other nucleases are involved in ribosome assembly. The endoribonuclease Las1, for example, mediates a critical step in the maturation of the 60S large ribosomal subunit that triggers the decay of the Internal Transcribed Spacer 2 (ITS2) sequence separating the 5.8S and 25S rRNA [1]. Depletion of Las1 leads to aberrant localization of ITS2-containing pre-60S subunits into the cytoplasm, which can enter the translation pool but causes translation defects [9]. Like miscleaved 18S-containing ribosomes, ITS2-containing ribosomes also recruit the RQC machinery to target these misprocessed ribosomes for decay. This suggests that recruitment of the RQC machinery could be a generalized safeguard for ribosome assembly to detect and remove improperly constructed ribosomal subunits. However, it will be important to establish if miscleavage by other ribosome assembly nucleases also promotes ribosome collision-mediated decay.

The ATPase Rio1 and nuclease Nob1 are major players in coordinating the final stages of SSU assembly, but many questions remain about how these assembly factors carry out their functions. One such question: could there be a benefit to Nob1 miscleavage? Parker and colleagues have established that Nob1 can miscleave in vivo and prefers to cleave RNA 5′ of adenosines, of which there are several in the vicinity of the ITS1 cleavage site [6]. Inspection of a previously determined cryo-EM structure containing Nob1 bound to its ITS1 and 18S 3′ end substrate shows weak electron density for this rRNA, suggesting putative conformational dynamics which could influence how Nob1 ultimately cleaves this substrate [5]. While ribosomes containing improper 18S 3′ ends are likely not advantageous and would be targeted for decay, this is a source of ribosome heterogeneity and could be used as a mechanism to alter rates of translation under conditions where collisions are limited. Healthy cells (especially in higher organisms with diverse cell types) that might be utilizing this altered translation rate productively would need to be identified and studied further.

Another open question: how does Rio1 inspect the 3′ end of the 18S? Cryo-EM structures of Rio1 bound to the pre-40S at a variety of states place Rio1 in the vicinity of h44 but do not show it interacting directly with the 3′ end [2–4]. Rio1 likely samples additional dynamic conformational states on the ribosome that are not visible by EM. Finally, are these quality control safeguards conserved across eukaryotes? Many steps of the ribosome assembly pathway are well conserved, but there are noted differences. Human pre-40S structures support the fact that, in addition to Rio1, binding of Rps26 and eukaryotic translation initiation like factor 1A domain containing protein (EIF1AD) may also help ensure proper 3′ end formation [3,4]. While more studies are needed, it seems plausible that higher organisms have additional mechanisms and/or alternative checkpoints to ensure fidelity during ribosome assembly.
